# Cases of Recovery from Wounds of the Brain

**Published:** 1834

**Authors:** K. Estep

**Affiliations:** Stark county, Ohio


### Art. V.—Wounds of the Brain.

*Cases of recovery from Wounds of the Brain, and from extensive comminuted Fracture of the Cranium.* By Dr. K. Estep, of Stark county, Ohio.

---

Our object in admitting the following cases, is to multiply the evidences that the brain may be deeply wounded, and the cranium shattered into many fragments, and the patients still live, without suffering any impairment of intellect. Ed.

---

Case 1.—June 23d, 1828—Was called to see the daughter of Thomas Falls, a little girl, eight years old, on whom a board, 5 feet long, 20 inches wide, and 1¼ inches thick, had fallen from the floor above. It had descended end-wise, and struck the child as she lay sleeping on the floor, in the left temple, when a marked depression and softness were observable; the one corner having struck the head immediately below the origin of the temporal muscle, the other probably the arm or side. The right cheek and temple were slightly excoriated from pressure, perhaps from a little sliding motion on the floor—General symptoms, such as are usual in cases of fractured skull—pulse regular, but slow—eyes half open, with permanently dilated pupils—insensibility—vomiting and stertorous respiration.

On the return of the parents, and their approbation being obtained, I proceeded to remove the scalp, by a semi-circular incision, embracing the depression. Turning down the flap,

I found that the intervention of the temporal muscle prevented me from coming at the fracture. Separating with a scalpel, a few of the first fibres of that muscle from the bone, I discovered the breach, and at the same time a quantity of brain and blood burst through the opening.

The extent of the fracture downward, I never ascertained; the depression being so great, that I never either saw or felt the fragments. The quantity of brain that escaped, must have exceeded a table spoonful; that of blood, 24 oz.

June 24th—Patient sensible—asks for, and receives food—bowels constipated—pulse slightly accelerated—gave saline aperients and injections.

25th—Patient tolerably easy, and perfectly sensible.

26th—Dressed the head—wound looks well—a large portion having healed by the first intention.

From this time forward, I did nothing more than apply the proper dressing, and administer aperients when necessary.

In two weeks, the external wound was entirely healed, with the exception of a small space, nearest the fracture, designedly left open, for the escape of whatever matter might accumulate—the child going about in tolerable health, in a fortnight—was entirely restored in three months.

This child, now about fourteen years old, is enjoying good health, and has intellectual powers, on a level with other members of the family.

Case 2.—Dec. 11th, 1830, Solomon Wiseman, 17 years of age, had his skull fractured by the falling of a tree. I was immediately called to the scene, about nine miles distant. On examination, found both the parietal and occipital portions of the cranium involved in the mischief.

The limb of the tree, which may have been 4½ inches, in diameter, had fairly imbedded itself in the upper part of the head. General symptoms, such as are usual under such circumstances—dilated pupils—stertorous breathing—slow pulse—insensibility—vomiting, &c. With little hope of a successful result, I made an incision, beginning at the right temple, and passing round immediately above the crucial spine of the occiput, terminated near the opposite temple. Turning over the flap, I exposed the whole superior portion of the skull, when the faint hope I might have cherished was extinguished. The whole arch of the cranium was shattered into fragments, the longest of which did not contain two square inches of whole bone, and from that down to half an inch of every variety of figure.

Instead of the natural vault of the skull, a deep concavity, corresponding with the cylindrical form of the limb, presented angles and spiculæ, pointing in every direction. At this stage of the operation, I made a pause, undecided whether to proceed, or close up the wound, and let him die in peace.

As there was yet vitality, and as even a dead body would look more seemly with something resembling a head (which then he had not) I decided on the former. And here no small difficulty presented; for had I removed every fragment, which separately contemplated would have seemed to require it, I should have left the upper portion of the brain, without a bony covering. Pursuing a middle course, therefore removing some fragments and replacing others, I succeeded in restoring the head to something like its original shape. It is worthy of remark, that on removing one fragment, consisting of parts of the right parietal and the occipital bones, the patient, for the first time since the accident, spoke. Of the quantity of blood lost, no proper estimate, in the crowd and confusion of the place, could be made. Many branches of the temporal artery externally, and those of the dura mater internally, were divided and torn up; all of which, however, ceased bleeding spontaneously. Having closed the wound and bandaged the head, I took my departure, with this injunction to the friends—that, if contrary to every rational hope, he should survive to the third day, then to send for me.

Dec. 21st. Contrary to my forebodings, a messenger arrived, requesting my attendance. Found the patient sensible. When asked who I was, answered correctly. Said he remembered my dressing his head, though his eyes were then and now hermetically sealed. Removed the applications and gave aperients, which, with simple dressing, and occasional bloodlettings, constituted the outline of the treatment, till June 2d, when a profuse hæmorrhage ensued, and recurred at intervals, but was always arrested by saturnine preparations, or digitalis.

May 22nd. The external wound is healed with but a trifling exception, from which a sanious discharge continues to distil—found a fragment of bone an inch long, half an inch wide, lifeless, and *partially dissolved*, keeping up irritation; enlarged the opening and removed it—found a bone deposited beneath, and permanent indentation of course remaining. Having removed this fragment, it is now before me, and the inner surface presents the appearance of having been preyed upon by some insect—being almost entirely eroded—the outer surface, but partially dissolved.

